# Structural Health Monitoring for a Z-Type Special Vehicle

**DOI:** 10.3390/s17061262

**Published:** 2017-06-01

**Authors:** Chaolin Yuan, Liang Ren, Hongnan Li

**Affiliations:** 1 State Key Lab of Offshore and Coastal Engineering, Dalian University of Technology, Dalian 116024, China; chaolinyuan@mail.dlut.edu.cn (C.Y.); hnli@dlut.edu.cn (H.L.); 2 School of Civil Engineering, Shenyang Jianzhu University, Shenyang 110168, China

**Keywords:** FBG sensors, special vehicle, structural health monitoring

## Abstract

Nowadays there exist various kinds of special vehicles designed for some purposes, which are different from regular vehicles in overall dimension and design. In that case, accidents such as overturning will lead to large economical loss and casualties. There are still no technical specifications to follow to ensure the safe operation and driving of these special vehicles. Owing to the poor efficiency of regular maintenance, it is more feasible and effective to apply real-time monitoring during the operation and driving process. In this paper, the fiber Bragg grating (FBG) sensors are used to monitor the safety of a z-type special vehicle. Based on the structural features and force distribution, a reasonable structural health monitoring (SHM) scheme is presented. Comparing the monitoring results with the finite element simulation results guarantees the accuracy and reliability of the monitoring results. Large amounts of data are collected during the operation and driving progress to evaluate the structural safety condition and provide reference for SHM systems developed for other special vehicles.

## 1. Introduction

Due to the rapid economic growth of China, the road transportation is undergoing a fast development period [1,2]. To satisfy specific transportation requirements, special vehicles are designed and produced differently from regular vehicles in overall size and appearance. The trend of large-scale and heavy-duty vehicles demands high structural stability. Accidents such as overturning will lead to large economical loss and casualties [3]. However, there have still been no technical specifications to follow to ensure the safe operation and moving process of special vehicles until now. Regular maintenance is of poor efficiency and usually carried out when the machines are in idle. Therefore, it is impracticable to determine their safety status while the machine is working. Due to the fact that special vehicles are usually under large loads, mechanical conditions will suffer many failures such as minor cracks and deformation of steel structures. In conclusion, it is necessary to conduct structural health monitoring (SHM) on special vehicles which can report the safety condition and locate the structural damage in real time.

Until now, the SHM system—widely applied to large-scale civil engineering structures such as bridges, airport terminals, and gymnasiums [4,5,6,7,8,9]—aims to develop automated systems for the continuous monitoring, inspection, and damage detection of structures with minimum labor involvement [10]. Generally, an effective SHM system is composed of three subsystems: a sensing system, data acquisition system, and data analysis system. The sensing system may use various kinds of sensors, the FBG sensor is one which has been extensively adopted as a new non-destructive evaluation technique in monitoring strain and temperature profiles of structures with smaller size and higher resolution [11]. The real-time monitoring for the safety condition of special vehicles using the FBG sensors with advantages mentioned above, will greatly improve safety situation instantly. In recent years, more and more studies have focused on the structural health monitoring of steel structure. Fatigue cracks on steel components of a structure may cause significant influence on the strength and serviceability. Henderson first carried out the monitoring of fatigue crack of a steel structure [12]; Ichinose and Lee used the FBG sensors to detect the structural failure caused by the cyclic loading [13]. Kharroub developed a smart sensing skin to detect and localize the fatigue cracks [14]. Kaloop et al. presented a movement analysis and assessment of a steel-contained crane based on structural health monitoring [15]; China Special Equipment Inspection Institute have made progress in large-scale hoisting machines’ structural health monitoring [16]. All these scientific achievements have laid a solid theoretical and practical foundation for the SHM of special vehicles. 

However, compared with cranes, special vehicles should perform the function of not only loading but also conveying. During transportation, road bumps can cause minor vibrations of the bodywork which is equivalent to impact load. Hence it is also important to monitor this process. In this paper, the SHM system has been conducted for a z-type special vehicle to monitor its whole working process—including loading, conveying, and unloading—continuously in real time. A finite element model is first built to simulate the force distribution to calculate the stress distribution during the lifting process. Then a reasonable SHM scheme is presented using the FBG sensors. Comparing the monitoring results with the finite element simulation results guarantees the accuracy and reliability of the monitoring results. Some conclusions at the end of this paper have been made to evaluate the safe condition during the operation and driving progress.

## 2. Z-Type Special Vehicle and FBG Sensors

**Figure 1:**
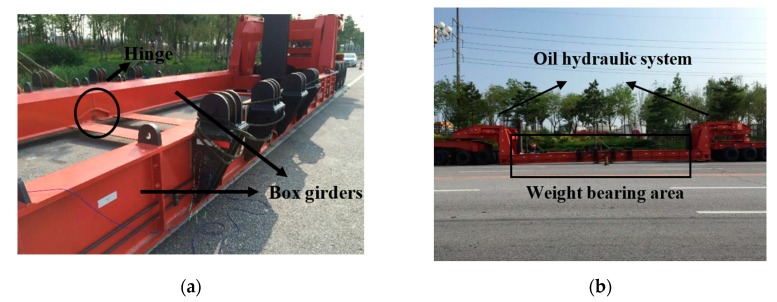
Overview of the z-type vehicle. (**a**) Hinge and box girders of the vehicle; (**b**) Oil hydraulic system and weight bearing area.

Figure 1 shows the entire structure of the z-type vehicle, where it can be seen that the structure is bilaterally symmetrical. The weight-bearing area is mainly composed of four box girders which are connected by several steel plates, and the black spools on the edge of the girders are used to fix cargo with wire cables. Box girders on the same side are hinged with each other. The oil hydraulic system, as labelled in Figure 1b, enables the weight-bearing area to rise up to fulfil the function of loading.

**Figure 2:**
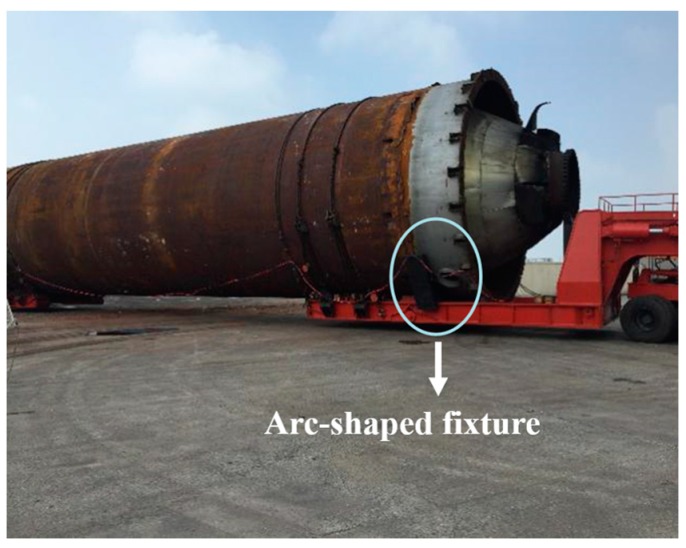
Weight bearing area separated at the hinge point.

The z-type special vehicle is required to transport a coke tower in a chemical plant, Figure 2. The 200-ton coke tower is placed on the circled arc-shaped fixture. The weight-bearing area needs to be separated in the hinge part to accommodate the 33 m-long tower.

**Figure 3:**
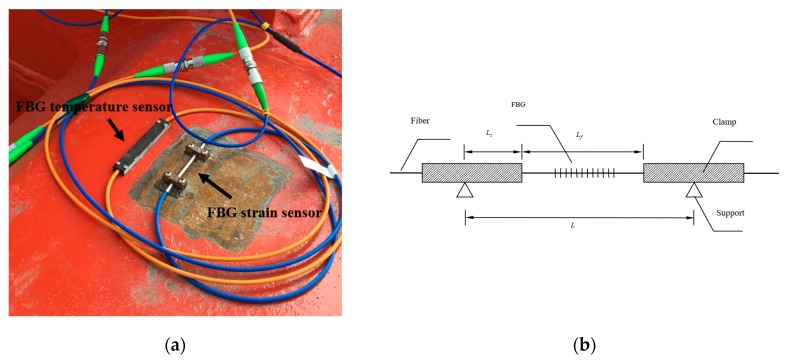
FBG sensors used in the structural health monitoring system. (**a**) FBG sensors; (**b**) Schematic of the clamp-package for FBG strain sensors.

The structural deformation would cause the stress change of the box girders of the vehicle such as the root of the weight-bearing area. The monitoring system in this case uses FBG strain sensors and FBG temperature sensors as the sensing system (Figure 3), developed by the structural health monitoring and control research center of Dalian University of Technology, to monitor the stress change of key members and to achieve temperature compensation respectively. The schematic of this clamp-package is shown in Figure 3b [17]. This package enables the fixture to be welded rather than glued on the structure which can eliminate the influence of strain transfer caused by adhesive. Furthermore, strain sensors can be much more easily installed and uninstalled.

Assuming that deformation Δ*L* appears between the two supports, then

(1) $$\Delta L=2\Delta L{\;}_{s}+\Delta L_{f}=2\frac{P_{s}L_{s}}{E_{s}A_{s}}+\frac{P_{f}L_{f}}{E_{f}A_{f}}$$

where, $\Delta L{\;}_{s\;}$ and $\Delta L{\;}_{f}$ are the deformation of the clamp and fiber respectively; $P_{s}$ and $P_{f}$ are the axial force of the clamp and fiber respectively; $E_{s}$ and $E_{f}$ are the young’s modulus of the clamp and fiber respectively; $A_{s}$ and $A_{f}$ are the sectional area of the clamp and fiber respectively.

As the axial force is all the same inside the package structure. Then

(2) $$\frac{\frac{\Delta L{\;}_{s}}{L{\;}_{s}}}{\frac{\Delta L_{f}}{L_{f}}}=\frac{\varepsilon_{s}}{\varepsilon_{f}}=\frac{E_{f}A_{f}}{E_{s}A_{s}}$$

where, $\varepsilon_{s}$ and $\varepsilon_{f}$ stand for the strain of clamp and fiber respectively.

Substitute the young’s modulus and sectional area into Equation (2) given in Table 1.

(3) $$\frac{\varepsilon_{s}}{\varepsilon_{f}}=0.0084$$

which means that the axial strain is mainly the fiber strain.

The strain of the structure ε can then be presented by

(4) $$\varepsilon =\frac{L_{f}∆\lambda_{FBG}}{1.2L}$$

where, $∆\lambda_{FBG}$ is the wavelength change of the FBG; $L_{f}$ is the distance of the clamps; L is the distance of the supports. 

The temperature compensation is realized by FBG temperature sensors which can measure the wavelength change caused by temperature. So, the mechanical strain of the structure $\varepsilon_{s}$ can be presented by

(5) $$\varepsilon_{s}=\varepsilon -\varepsilon_{T}$$

where, ε is the structure strain before temperature compensation and $\varepsilon_{T}$ is the strain measured by temperature sensors.

## 3. Sensor Layout Scheme

**Figure 4:**
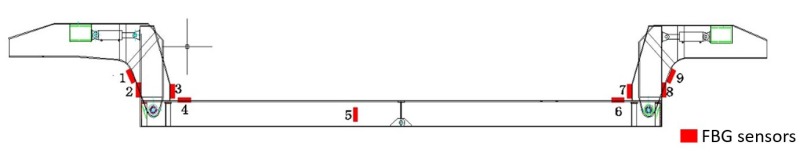
Layout scheme of the sensors.

In Figure 4, the arrangement of sensors is laid out. The sensors’ positions are the same as on the other side of the vehicle. The strain sensors and their initial wavelengths are provided in Table 2, in which the prefix “L” stands for the sensors installed on the left side and “R” for those on the right side of the vehicle, respectively. The signal of R1 sensor is lost during the whole process so in Table 1 its initial wavelength is “NaN”. 

In the following data analysis, the data from some of the sensors have been excluded because the stress changes are not obvious.

## 4. Monitoring Results of Vehicle’s Operating Process

The material of the main structure of vehicle is Q345 grade steel with the design strength of 295 MPa for safety reasons referring to the specifications.

### 4.1. Monitoring Results of Loading Process

**Figure 5:**
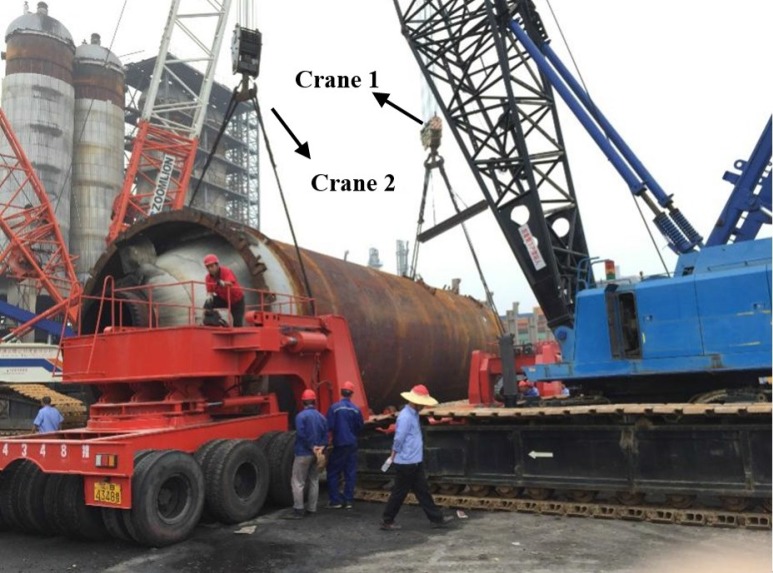
The hoisting work process.

Arc-shaped fixtures were first mounted on the weight-bearing area of the vehicle to fix the coke tower. After that, the coke tower was hoisted to the vehicle using two cranes (Figure 5).

The whole hoisting work lasted for 38 min. It occurred as follows:

1. The weight-bearing area rose up to a certain height.
2. The position of the tower was adjusted by two cranes.
3. The tower was hoisted slowly onto the fixtures until it was stable.
4. The tower was tied up using wire cable.
5. The weight-bearing area descended.

**Figure 6:**
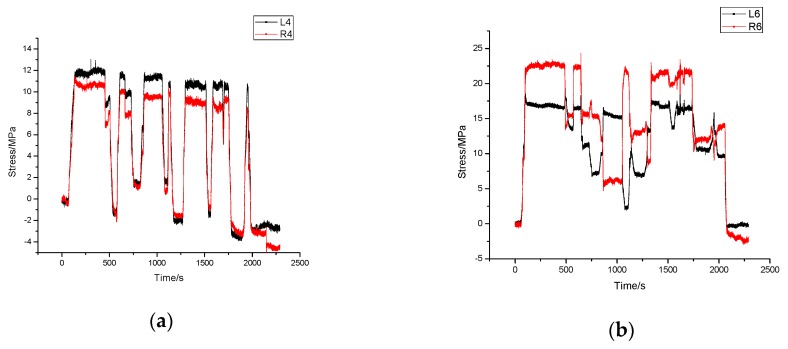
Stress time history diagram of the hoisting work. (**a**) Stress time history of sensor L4 and R4; (**b**) Stress time history of sensor L6 and R6.

In Figure 6, the stress time-history diagrams of sensor 4 and sensor 6 on both sides are given. The stress changes fluctuates which is caused by fine adjustment of the position of the tower when it came into contact with the fixture. It can be seen in Figure 6 that the maximum stress of sensor 6 is slightly larger than that of sensor 4 and, as the result of that, one end of tower reached the fixture first which is near to the location of sensor 6. 

During this process, the maximum stress change is 24.28 MPa, much smaller than the yield strength 295 MPa which means the whole structure is in safe condition.

### 4.2. Monitoring Results of Lifting Process 

As the hoisting work is conducted under the aid of two cranes, a test is then carried out to monitor the stress changes when the weight-bearing area is raised up.

**Figure 7:**
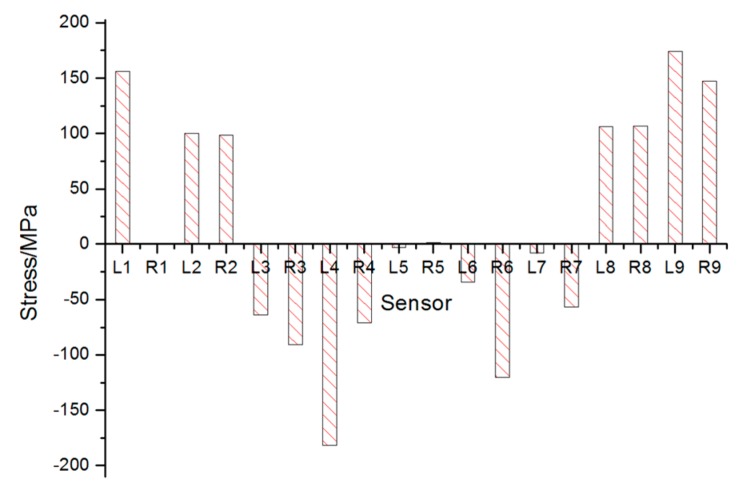
Stress distribution measured by all sensors during lifting process.

**Figure 8:**
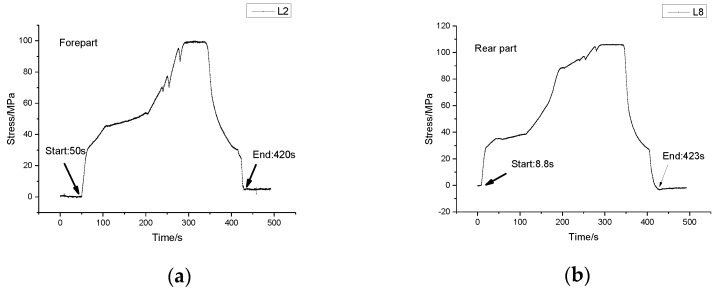
Stress time history diagram of sensors on the same side during lifting process. (**a**) Stress time history of sensor L2; (**b**) Stress time history of sensor L8.

Conclusions can be made as follows:

1. Figure 7 gives out the maximum stress changes measured by all sensors. It is obvious that sensors on both ends of the vehicle were in tension while those in the middle part in compression. The stress change of sensor 5 was rather small but noticeable. Monitoring result of sensor L7 is unexpectedly small due to the fact that the sensor is connected incorrectly.
2. The maximum stress of each sensor is shown in Table 3. In Table 3, the maximum compressive stress change 181.82 MPa occurred at L4 near to the root of the weight-bearing area as expected, while the largest tension stress change obtained from sensor L9 was 174.08 MPa. The monitoring area corresponding to the sensors set in bold in Table 3 should be reinforced for security reasons.
3. The last column of Table 3 is the stress differences between sensors on the left side and those on the right side. The 110.93 MPa stress difference between sensor L4 and R4 cannot be ignored, which indicated that stress was distributed unevenly on different side. So, the process of lifting goods on the weight-bearing area deserved more attention in the case of overturning.
4. During the lifting process of the weight-bearing area, the forepart and the rear part of the vehicle were not raised up at the same time (see Figure 8). The forepart was lifted up about 40 s later than the rear part. In that case, the rear part would bear most of the weight at the beginning of the process.

#### Finite Element Analysis

A finite element model was built to simulate the lifting process assuming that the vehicle structure was in elastic state to compare with the monitoring results using the ANSYS software. Shell element was adopted to simulate the structure.

Relevant parameters for the finite element model are listed in Table 4.

**Figure 9:**
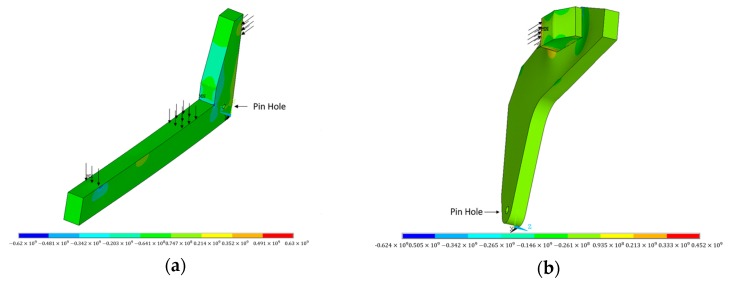
Finite element model of the special vehicle. (**a**) Loading arm; (**b**) shear leg.

Since the structure of the vehicle is symmetric, one-fourth of which is taken to build the finite element model as shown in Figure 9. All degrees of freedom have been constrained around the pin hole.

**Figure 10:**
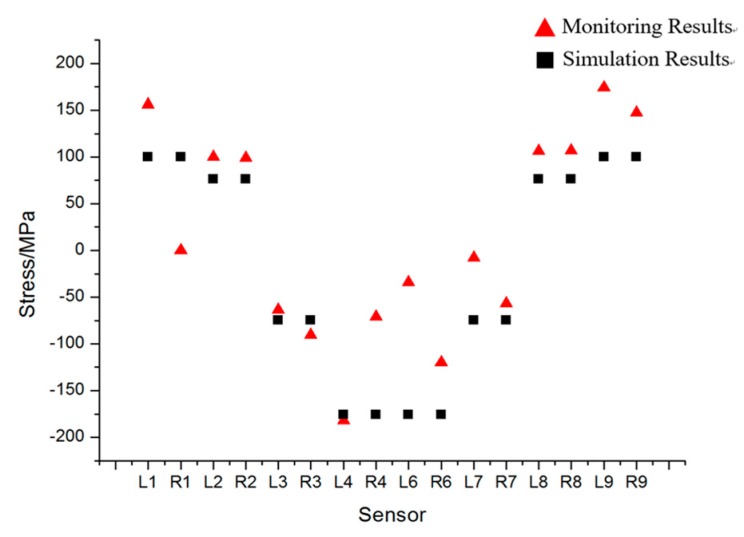
Simulation results comparing with the monitoring results.

In Figure 10, the simulation results are close to the monitoring results of sensor L2, R2, L3, R3, L4, R7, L8, and R8. However, differences of the rest sensors are large, which are acceptable on account of the finite element model being simplified, especially on the boundary conditions. The comparison results validate that the monitoring results are reliable.

### 4.3. Monitoring Results of Transportation Process 

During the transportation process, sensor data obtained under two special cases are analyzed as follows.

#### 4.3.1. Turning Process

**Figure 11:**
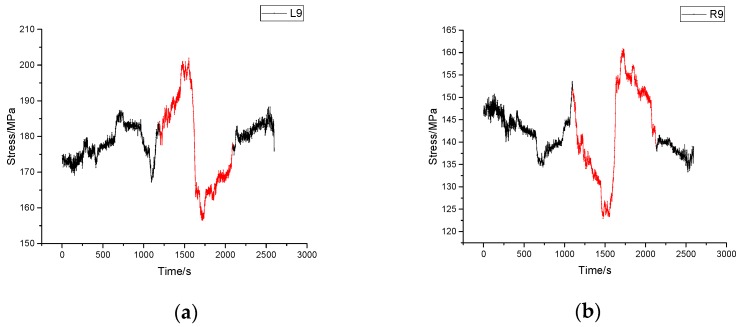
Stress time history diagram of turning process. (**a**) Stress time history of sensor L9; (**b**) Stress time history of sensor R9.

Figure 11 gives the stress time-history diagram of sensor 9 on both sides during the turning process. It is noteworthy that in this period, the stress changes of sensors on the left side and those on the other side vary in the opposite way (marked in red in Figure 11). When the vehicle began turning right, the stress of left-side increased while that of right-side decreased. By the end of the turn, the stress of all sensors was restored to previous level. In spite of the low velocity of the vehicle, the tower was so heavy that the vehicle slightly leaned to its right side. Hence it is easy to figure out that the left structure takes less weight of tower than the right. As it can be seen in Figure 11, the largest stress change is 195.73 MPa which indicates that the structure is in safe condition.

#### 4.3.2. Monitoring Results of Process of Passing through a Bump

**Figure 12:**
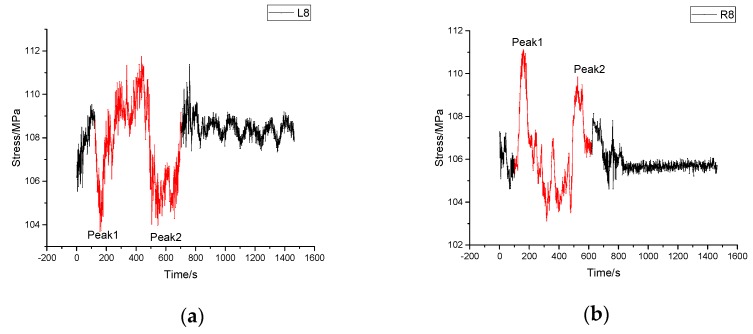
Stress time history diagram of passing through a bump. (**a**) Stress time history of sensor L8; (**b**) Stress time history of sensor R8.

The vehicle went via a bump with section height of 3 cm. The stress time-history diagram is shown in Figure 12. There are obviously two peaks. Peak 1 is caused by vibration when front wheel of the vehicle passed the bump. In the same way peak 2 is due to the vibration when back wheel passed the bump. Comparing the left-side sensor signals with those on the right side, they trend in an opposite way because the vehicle took a turn at the same time. During this process, the monitoring point of sensor 9 experienced the largest stress change of 208.58 MPa. In that case, the structure is safe.

### 4.4. Monitoring Results of Unloading Process 

**Figure 13:**
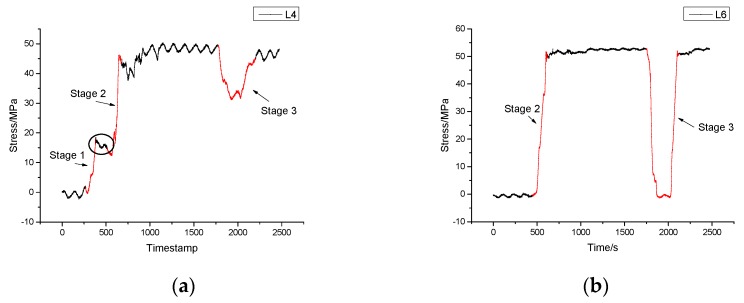
Mean stress time history diagram of the unloading process. (**a**) Stress time history of sensor L4; (**b**) Stress time history of sensor L6.

The unloading process was also carried out with the aid of two cranes. Figure 13 shows the mean stress time-history diagrams of the sensors. The unloading process could be divided into three stages and the stress change of the sensors mounted on the front part of the vehicle was a little different from those on the rear part.

The unloading process follows three stages as illustrated in Figure 13.

1. In the first stage, crane 1 first lifted up the forepart of the tower while the rear part was still in contact with the fixture. Then the lifted end of the tower was halted in the air which explained the platform circled in the diagram of sensor L4.
2. In the second stage, both of the cranes began to lift the entire tower up together.
3. In the third stage, crane 2 needed to adjust its position so that the tower was put down. The rear part of the tower was in contact with the fixture again while the forepart was still lifted by crane 1, which explained the difference of magnitude in stage 3 between sensor L4 and L6.

Conclusions can be drawn in this process as follows.

1. In Table 5, the maximum stress of each monitoring point is given out in the process of unloading. The differences in magnitude between the strain levels and the directions of change are dependent on sensor location. It is obvious that sensors on both ends of the vehicle were in compression while those in the middle part were in tension which was the opposite of the expected lifting process.
2. Differences between sensors on the left side and the right side are quite small. The maximum stress 53.83 MPa appeared in R6 which indicated that the structure was in safe condition.

**Figure 14:**
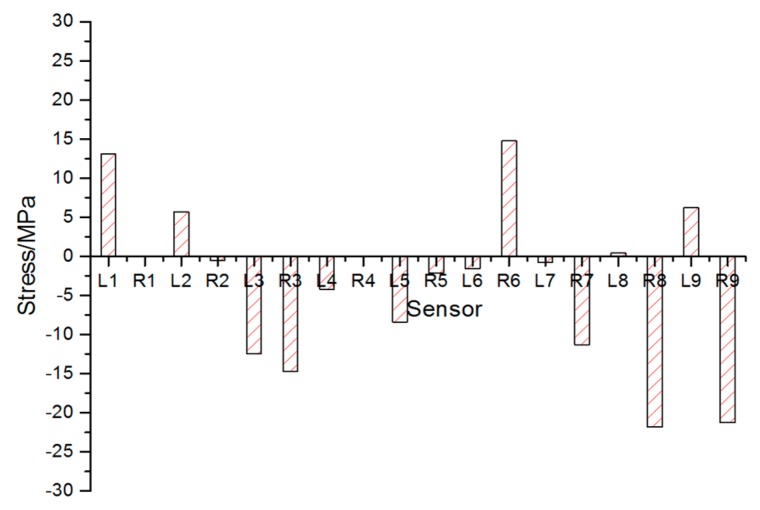
Residual stress measured through all sensors.

In Figure 14, the total stress changes of each monitoring point were given out calculated by the difference between the wavelengths of sensors after the unloading process and the initial wavelength. The wavelengths of some sensors were not restored to their initial status which was due to the fact that the fixtures still stayed on the vehicle and the weight-bearing area did not descend.

## 5. Conclusions

In this paper, the SHM applied to a z-type special vehicle is introduced—including the scheme of sensor location, finite element modeling, and data analysis—which realized real-time monitoring of the whole working process and guaranteed the safety transportation. Some conclusions can be made as follows:

1. The scheme of sensor location is presented which can provide a reference for other similar structures of special vehicles.
2. Comparing the result of finite element analysis and that of real monitoring makes the SHM for the vehicle reliable.
3. Data analysis guarantees safety for the vehicle operating process during which the sensors can monitor the structural responses in real time. It can be assumed that structural damages can be detected in time to prevent accidents.

## Figures and Tables

**Table 1:** Mechanical properties of the optical fiber.

Parameter	Magnitude	Unit
$E_{f}$	$7.2\times 10^{10}$	Pa
$E_{s}$	$210\times 10^{9}$	Pa
$A_{s}$	0.50	mm²
$A_{f}$	0.01	mm²

**Table 2:** Sensor number and wavelength.

Sensor Number	L1	L2	L3	L4	L5	L6	L7	L8	L9
Initial Wavelength (nm)	1515.740	1521.486	1537.313	1545.061	1553.702	1561.428	1569.029	1577.231	1585.110
Sensor Number	R1	R2	R3	R4	R5	R6	R7	R8	R9
Initial Wavelength (nm)	NaN	1529.667	1536.998	1545.519	1553.209	1561.886	1569.350	1577.896	1584.892

**Table 3:** The maximum stress of each monitoring point.

Sensor Number	Max Stress/MPa	Sensor Number	Max Stress/MPa	Difference/MPa
L1	156.03	/	/	/
L2	100.01	R2	98.78	1.23
L3	−63.62	R3	−90.66	27.04
L4	−181.82	R4	−70.89	110.93
L5	−2.7	R5	1.62	1.08
L6	−34.29	R6	−119.97	85.68
L7	−8.06	R7	−56.76	48.7
L8	106.24	R8	106.73	0.49
L9	174.08	R9	147.28	26.8

**Table 4:** Parameters of finite element model.

Element Type	Density	Young’s Module
Shell element	7850 kg/m^3^	210 GPa

**Table 5:** The mean maximum stress change of each monitoring point.

Sensor Number	Max Stress/MPa	Sensor Number	Max Stress/MPa	Difference/MPa
L1	−35.98	-	-	-
L2	−22.82	R2	−23.39	0.57
L3	22.19	R3	26.95	4.76
L4	50.31	R4	50.87	0.56
L5	5.54	R5	2.21	3.33
L6	53.13	R6	53.83	0.7
L7	2.25	R7	16.79	14.54
L8	−22.27	R8	−21.83	0.44
L9	−44.94	R9	−35.49	9.45
